# The Utility of Microbial Cell-Free DNA in the Rapid Diagnosis of Chagas Disease: Chagas Disease Case Series

**DOI:** 10.1093/ofid/ofag584

**Published:** 2026-10-12

**Authors:** Anissa Amilhamja, Adam Shaikh, Kristin Mondy, Michael Kron, Niki Viradia, Omer Beaird, Jaime Fergie

**Affiliations:** Department of Pediatrics, Driscoll Children's Hospital, Corpus Christi, Texas, USA; Department of Pediatrics, Driscoll Children's Hospital, Corpus Christi, Texas, USA; Division of Infectious Diseases, Dell Medical School, Austin, Texas, USA; Department of Infectious Disease, Froedtert and Medical College of Wisconsin, Milwaukee, Wisconsin, USA; 4th Year Medical Student, Froedtert and Medical College of Wisconsin, Wauwatosa, Wisconsin, USA; Department of Infectious Disease, UCLA, Los Angeles, California, USA; Department of Infectious Disease, Driscoll Children's Hospital, Corpus Christi, Texas, USA

**Keywords:** chagas disease, global health, karius, mcfDNA, prolonged fever

## Abstract

**Background:**

Chagas disease (CD) is a leading tropical disease and the most prevalent parasitic infection in Latin America, affecting >7 million people globally and ∼240 000–350 000 individuals within the United States. Though known to be endemic to Latin America, prevalence in the United States is also increasing with improved recognition of locally acquired cases, especially in Texas. Autochthonous cases occur throughout the Southern United States, but the geographic range is anticipated to shift northwards as a result of climate change. Chagas disease is referred to as a “silent and silenced disease” by the World Health Organization as most people show no signs or symptoms for years following acute infection, and it primarily affects individuals who are socioeconomically disadvantaged. Historically, Chagas disease has been underrecognized. In the absence of treatment, infected individuals progress to a clinically silent or “indeterminate form” of infection that persists for life. While most patients with indeterminate Chagas disease do not have long-term sequelae, ∼30%–40% will develop end-organ cardiac or gastrointestinal disease decades after infection. Due to its protean clinical presentations, diagnosis requires a high clinical index of suspicion and knowledge of the epidemiology. Traditionally, the diagnosis is based on serology, visualization of the parasite in blood, and polymerase chain reaction (PCR), all of which require suspecting the infection. Microbial cell-free DNA (mcfDNA) of blood can serve as a useful tool for detection of *Trypanosoma cruzi* even when Chagas disease is not suspected.

**Results:**

Here we present 7 cases, 3 children and 4 adults, who presented with nonspecific manifestations; CD was not in the initial differential diagnosis in 5 of them. Chagas disease was ultimately diagnosed by Karius Spectrum test. In 1 case, the test was sent to expedite diagnosis in the setting of suspected Chagas reactivation.

Also known as American trypanosomiasis, Chagas disease is caused by *Trypanosoma cruzi*, a hemoflagellate protozoa that utilizes triatomine bugs as its primary vector. These “kissing bugs” are active at night, feeding on human and animal blood. Infection has 3 distinct stages. The acute phase of infections lasts between 4 and 8 weeks and is associated with nonspecific symptoms including prolonged fever, panniculitis, and a morbilliform rash. Severe manifestations, such as acute myocarditis, may also occur, with mortality ranging from 0.2% to 0.5% [1]. In the absence of treatment during the acute phase, infection persists for life. Thirty percent to 40% of individuals with chronic Chagas disease may develop a series of complications, such as cardiomyopathy, megacolon, and stroke [2]. Chagas cardiomyopathy is a leading indication for heart transplantation among people from endemic countries and has been associated with high rates of reactivation in the context of transplant immunosuppression [3]. Children are more symptomatic, with higher mortality rates during the acute phase, but demonstrate better treatment success compared with adults for reasons unknown [1].

Diagnosis has classically relied on peripheral blood smear (PBS) and PCR during acute infection and immunoglobulin (Ig) G/IgM serologic studies, such as enzyme-linked immunoassay (ELISA), indirect hemagglutination test (IHA), or indirect immunofluorescence (IIF) for chronic infection [4]. Recently, cases of acute, locally acquired Chagas disease have been diagnosed with the use of commercially available mcfDNA in patients where the diagnosis was not otherwise initially considered. To our knowledge, there is only 1 case report that described the reactivation of Chagas disease in a 50-year-old woman diagnosed with the use of mcfDNA or Karius Spectrum test [5]. The Karius Spectrum test has proven to be a powerful tool in identifying infectious etiologies in complex illnesses with a broad differential diagnosis, such as culture-negative infective endocarditis or fever of unknown origin. The test detects and quantifies cfDNA in plasma for a wide range of viruses, bacteria, fungi, and parasites. It is an innovative test that has made a landmark in the infectious disease world since 2017 [6]. Dedicated PCR-based tests typically have higher sensitivity. However, the main benefit of cfDNA metagenomic next-generation sequencing (mNGS) assays like Karius is their ability to quickly detect a potentially broad range of pathogens.

## CASE 1: CHAGAS CARDIOMYOPATHY

A previously healthy 12-month-old male infant who resided in Mexico for the first 3 months of life was transferred to Driscoll Children's Hospital, Corpus Christi, Texas, after a 6-day history of subjective fever, decreased level of activity, and anorexia. At the referring facility, he was found to have cardiomegaly, anemia, leukocytosis, and elevated troponin and brain natriuretic peptide (BNP) levels.

Upon arrival, he was found to be in respiratory failure and was intubated. A grade 2/6 systolic murmur was present along with hepatomegaly. Echocardiogram identified severe left ventricular (LV) dysfunction with ejection fraction (EF) of 30%–35%, a thrombus in the left ventricle, and pericardial effusion (Figure 1).

**Figure 1.**
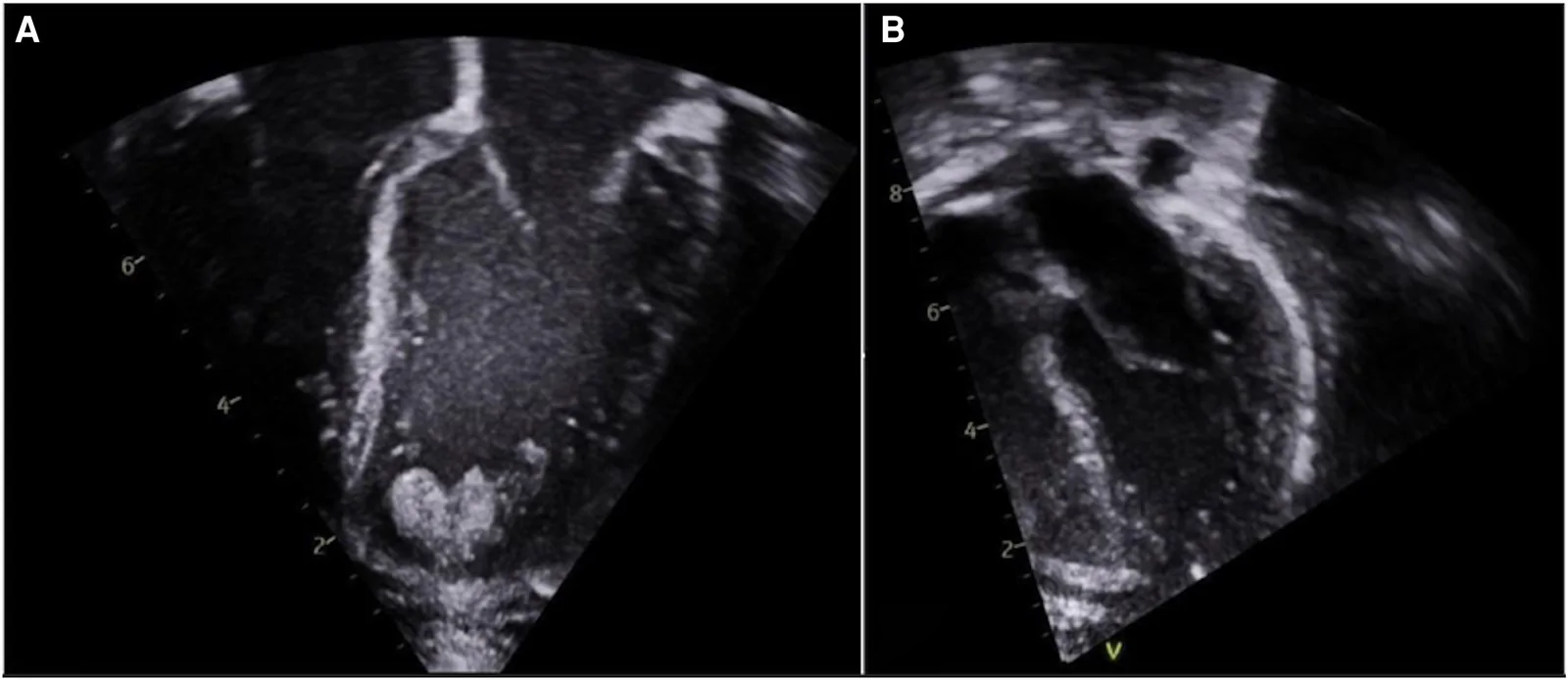
Echocardiogram of a 12-month-old male showing thrombus. A, Thrombus in the left ventricle apex measuring 6×7 mm and 6×6 mm, bilobed or 2 adjacent clots. Small to moderate pericardial effusion. B, Resolution of left ventricle thrombus.

Coronavirus disease 2019 (COVID-19) IgM antibody was positive. He was found to have an elevated C-reactive protein (CRP), prolonged prothrombin time (PT), and partial thromboplastin time (PTT) and low fibrinogen, transaminitis, and hypoalbuminemia. He was diagnosed with heart failure and dilated cardiomyopathy caused by myocarditis and hemophagocytic lymphohistiocytosis (HLH).

A Karius test was ordered on the second day of admission; it detected nonquantifiable *T. cruzi* 48 hours after the sample was collected. Bone marrow aspiration was performed, with PBS ordered on the third day of admission, which showed *T. cruzi*. Benznidazole was started. A second Karius test obtained 3 weeks after the first one, once HLH resolved, reported *T. cruzi* at 198 molecules/µL. The patient improved. EF and ventricular function slowly normalized, with weekly echocardiograms, and HLH resolved. There was no documented follow-up after discharge.

## CASE 2: PEDIATRIC MYOCARDITIS SECONDARY TO CHAGAS DISEASE

An 11-month-old female infant with a history of recurrent otitis media and thyroglossal duct cyst was admitted for elective bilateral myringotomy tubes and resection of thyroglossal cyst. While in the operating room, she experienced cardiac arrest after myringotomy tube insertion. Cardiopulmonary resuscitation (CPR) was performed for 2 minutes with return of spontaneous circulation. The patient was intubated and placed on mechanical ventilation. Before the event, the patient had a decrease in appetite and level of activity for the past 2 weeks, in addition to swelling of the lower extremities, that was suspected to be related to insect bites. Epidemiological history includes living in a home in a rural town in South Texas. Mother was born in the United States with no known history of Chagas disease. Family and social history and travel history were unremarkable.

The chest x-ray was concerning for cardiomegaly. An echocardiogram revealed trivial pericardial effusion with mild tricuspid and mitral valve regurgitation. However, baseline troponin and BNP levels were elevated, leading to a follow-up echocardiogram the following day, which showed worsening pericardial effusion and systolic function with a left ventricular EF at 45%–49%. The patient was diagnosed with myocarditis. A Karius test was ordered. Two days later, the test was positive for *Trypanosoma cruzi* at 95 996 DNA molecules/uL and parvovirus. PBS was also collected and was positive for *T. cruzi.* Inflammatory markers were elevated. Blood culture had no growth, along with an unremarkable complete blood count (CBC). Additional labs were ordered to rule out other infectious processes, such as enterovirus, and were unremarkable. The child was then diagnosed with myocarditis secondary to *T. cruzi* and was started on a 60-day course of benznidazole. She was transitioned to room air, with improvement in troponin levels and systolic function on repeat echocardiogram a week later. She was closely followed outpatient by the infectious disease department. After treatment, a repeat *T. cruzi* PCR was positive, leading to weekly collections of PCRs sent to the Centers for Disease Control and Prevention (CDC) and microbiology lab. She was then restarted on antiparasitic medication with nifurtimox. She had 1 more positive result followed by 3 equivocal results until CDC PCR was finally negative, and the medication was then discontinued.

## CASE 3: CARDIAC FAILURE SECONDARY TO CHAGAS DISEASE

A previously healthy 9-month-old male infant presented at the emergency department (ED) with fever and decreased oral intake. About 3 weeks before admission, he experienced intermittent febrile episodes with cough. After the fever resolved, he developed an erythematous generalized exanthem that lasted about 2 weeks. According to the family, his symptoms began after his brother was diagnosed with COVID-19 infection. However, the patient was not tested for COVID-19. After several days of being afebrile, he was noted to have subjective fever and cough now associated with watery diarrhea and nonbilious emesis. He also had eye puffiness, tongue swelling, and a polymorphic rash.

He was taken to the ED, where he was diagnosed with an upper respiratory infection, and was discharged with close observation. He showed brief improvement for a few days until he experienced febrile episodes with an associated decrease in oral intake, urine output, and activity. Initial impression was presumed urinary tract infection (UTI) due to positive leukocyte esterase with pyuria along with leukocytosis. Lactate dehydrogenase was markedly elevated along with procalcitonin. The patient was given a dose of ceftriaxone for presumed UTI. A multiplex respiratory panel (SpotFire) was negative. He was then admitted for intravenous antibiotics and hydration. A chest x-ray revealed cardiomegaly with pleural effusion. A junctional rhythm was seen on his EKG, and a moderate concentric left ventricle hypertrophy, dyssynchronous ventricular contraction, and small pericardial effusion were noted on his echocardiogram. Troponin and BNP levels were elevated. He was transferred to the intensive care unit (ICU) with suspicion for potential myocarditis or possible multisystem inflammatory syndrome in children (MIS-C) secondary to COVID-19 infection. Intravenous immunoglobulin (IVIG), steroids, and diuretics were started. A Karius test was ordered along with a PBS. Both were positive for *T. cruzi*. The patient was then diagnosed with acute Chagas myocarditis, and benznidazole was started. In the cardiac ICU, his cardiac function deteriorated, with increases in troponin and BNP levels, and he was intubated. A follow-up echocardiogram showed a thrombus in the apex of his left ventricle and biventricular dysfunction with an EF of 20%. Due to severe cardiac dysfunction and associated ventricular arrythmia in the setting of Chagas disease, the patient was placed on extracorporeal membrane oxygenation (ECMO) support, and he was transferred for possible use of a left ventricular assist device and evaluation for heart transplantation. At the referring facility, he continued antiparasitic medication. He improved and was decannulated with later extubation. One month after discharge, he was seen for follow-up by the infectious disease department, where *T. cruzi* PCR was collected 1 week before completion of treatment. The test was negative for Chagas disease.

According to the parents, their family lives on a farm. They have had problems with fleas in the past. However, there is no travel history or history of Chagas disease.

## CASE 4: CENTRAL NERVOUS SYSTEM REACTIVATION WITH CHAGAS DISEASE

An 88-year-old woman with a history of hypertension, transient ischemic attack, and rheumatoid arthritis on methotrexate was admitted to Froedtert Hospital, Wisconsin, due to progressive altered mental status and right-sided weakness over several months. The patient was born and lived in Lima, Peru, before immigrating to the United States in 1963 at 34 years old and had not traveled back for >20 years. In the 2 years before this hospital admission in Milwaukee, Wisconsin, she lived in Oklahoma and shuttled between her daughters' homes in Wisconsin and Texas. The patient's family reported that her neighbor in Oklahoma had many cats that would stray into her backyard. She has otherwise never had any pets nor lived or worked on a farm while in the United States.

Brain magnetic resonance imaging demonstrated bilateral frontoparietal enhancing white matter changes and surrounding vasogenic edema (Figure 2). Brain imaging suggested an inflammatory process such as acute demyelinating encephalomyelitis (ADEM), tumefactive demyelination, or lymphoma, but an atypical infection could not be excluded. Cerebrospinal fluid (CSF) analysis demonstrated pleocytosis with 24 WBCs/µL with 88% lymphocytes and 12% monocytes. CSF protein was elevated at 146 mg/dL, and glucose was normal at 63 mg/dL. PBS for parasites was negative. The patient underwent a brain biopsy that demonstrated necrotizing encephalitis and abundant intracellular protozoa. Karius revealed 40 000 *T. cruzi* DNA molecules/µL. CSF and DNA extracted from formalin-fixed, paraffin-embedded brain tissue were both positive for *T. cruzi* DNA by PCR at the CDC.

**Figure 2.**
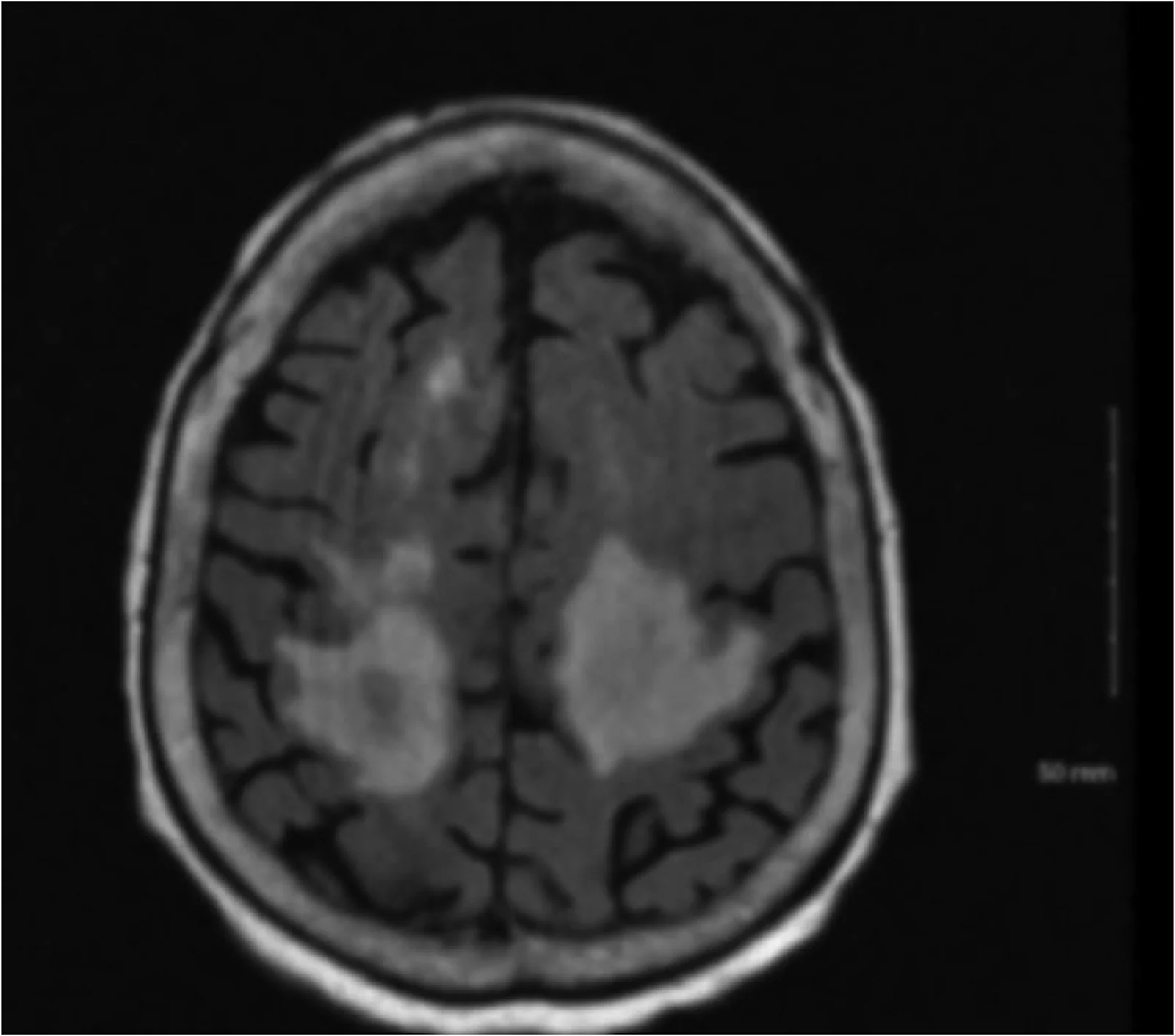
Brain magnetic resonance image of an 88-year-old female with necrotizing encephalitis demonstrating bilateral frontoparietal enhancing white matter changes and surrounding vasogenic edema.

The patient was started on benznidazole; however, despite aggressive supportive care, the patient died after 1 month. Serial PCR studies of the CSF over 2 weeks did not show clearance of *T. cruzi* DNA.

## CASE 5: ACUTE CHAGAS IN CENTRAL TEXAS

A 71-year-old woman with type 2 diabetes presented to Dell-Seton Medical Center in Austin, Texas, with complaint of intermittent fever, chills, dry cough, and rhinorrhea for the past 10 days. She had tested positive for COVID-19 by a home test 9 days prior and had completed a 5-day course of Paxlovid but continued to feel unwell. In the ED, her temperature was 102.7°F, with elevated heart rate and blood pressure. She had leukocytosis and elevated IF and was admitted for sepsis.

Her epidemiological history revealed that she had been camping in a nearby wooded area near Austin, Texas, about 3 weeks prior and had likely sustained a bug bite on her upper right arm that developed into a large, raised, circular area of erythema extending up to 5 cm in diameter (Figure 3). A relative who is an entomologist had concerns about a possible triatome bug being the cause of the bite. About a week after the bite, she sought consult with her primary care doctor, who performed a commercial *T. cruzi* IgG, and she was prescribed a 7-day course of trimethoprim-sulfamethoxazole for possible arm cellulitis. IgG for *T. cruzi* was reported at a titer of 1:8. However, this test was viewed as a possible false-positive result based on the low titer.

**Figure 3.**
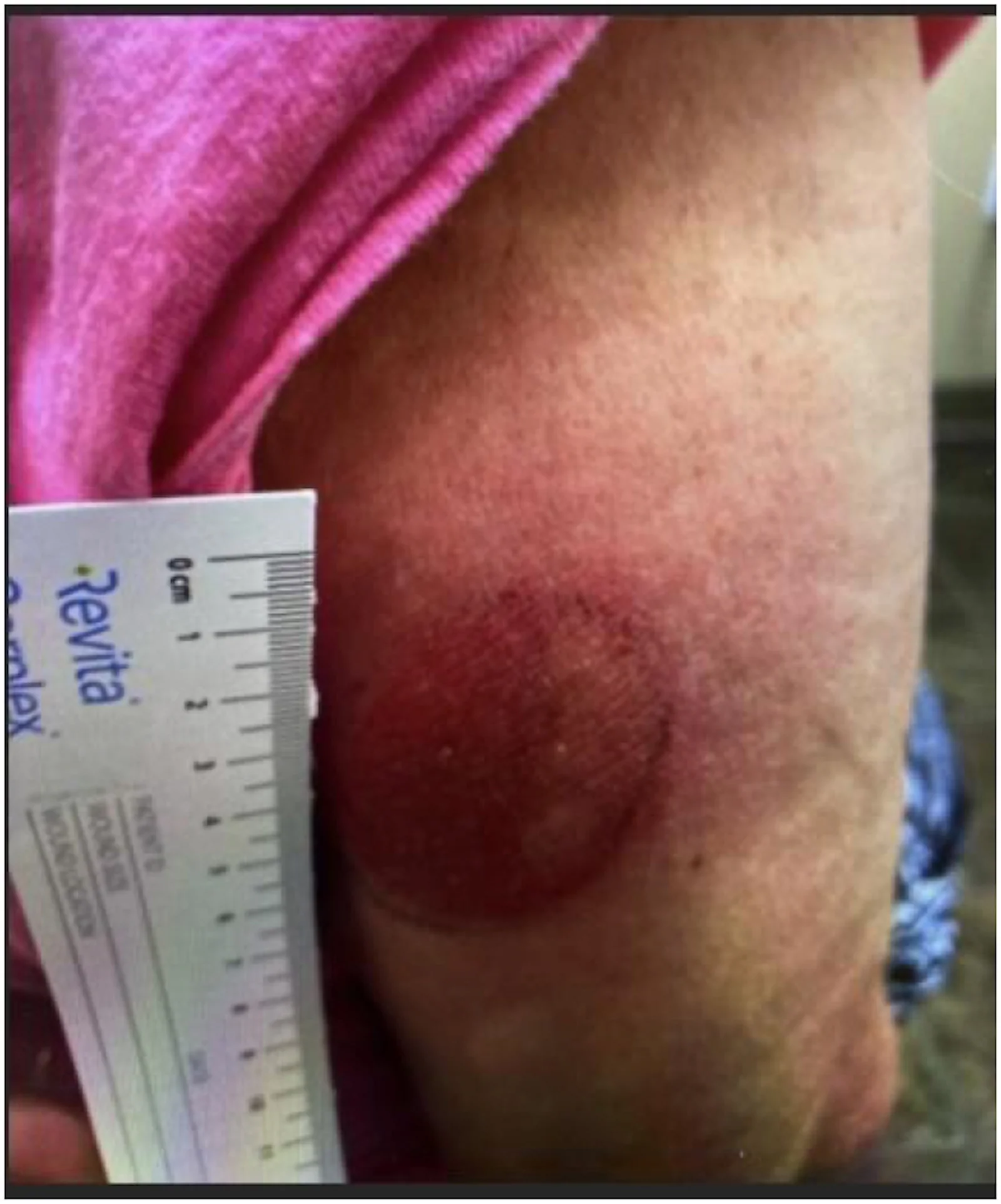
Chagoma on upper right arm of a 71-year-old female presenting as a raised, circular erythematous lesion (∼5 cm).

Following hospital admission, 2 sets of blood cultures and a PBS were obtained and were negative. COVID-19 PCR test was positive. Despite a negative PBS and possible inaccuracy of the *T. cruzi* IgG test, there was still a high suspicion of Chagas disease based on her history. A *T. cruzi* PCR test was expected to take about 2 weeks to result; hence a Karius test was ordered. A Karius test was sent on hospital day 2 and on day 4 reported detection of *T. cruzi* at 1379 DNA molecules/µL. A confirmatory *T. cruzi* PCR test was sent to the CDC and was also positive. Due to lack of availability of benznidazole, the patient was treated with nifurtimox, which she tolerated well. Repeat *T. cruzi* PCR was negative at follow-up. To date, she has had no further long-term sequelae of Chagas disease.

## CASE 6: OCULAR CHAGAS

A 41-year-old man with no significant medical history was transferred to a multispecialty hospital for worsening peri-orbital erythema, edema, and fever despite broad-spectrum antibiotics. Approximately 2 weeks prior, he recalled having a foreign-body sensation in his left eye after working outdoors at his ranch (∼30 miles south of Austin, TX, USA).

Over the following 2 days, he developed mild conjunctival injection with periorbital erythema and edema but no vision loss, fever, or constitutional symptoms. His eye symptoms worsened, and on day 5 of illness he saw an ophthalmologist, who hospitalized the patient out of concern for orbital cellulitis. He was started on empiric intravenous vancomycin and piperacillin/tazobactam. Testing for HIV, syphilis, and methicillin-resistant *Staphylococcus aureus* nasal colonization were negative. On day 8, he developed a palpable left preauricular lymph node, prompting a switch to intravenous rifampin and doxycycline for possible *Bartonella* oculoglandular syndrome. *Bartonella* serologies and blood cultures were negative. On day 11 of illness, the patient became febrile. His antibiotics were further broadened to include vancomycin, cefepime, and acyclovir, and on day 15 he was transferred to a tertiary care center.

Upon transfer, he was noted to have a temperature of 101.4°F (orally) and mild tachycardia with normal blood pressure and respiratory rate. His physical exam was notable for left periorbital edema, erythema, conjunctival injection (Figure 4), and the preauricular lymph node. He had no ocular pain or limitations of extra-ocular movements. A magnetic resonance image (MRI) of the orbit demonstrated enhancement of left medial and inferior rectus muscles, compatible with orbital myositis. Laboratory studies demonstrated a normal CBC and comprehensive metabolic panel. Additional tests were sent for toxoplasmosis, tularemia, and adenovirus (all negative) as well as a Karius test. On day 16 of illness, he was started on steroids for possible noninfectious etiologies, with improvement in his symptoms. He was discharged the subsequent day on a steroid taper. Following discharge, the Karius test resulted positive for *T. cruzi* at a level of 505 DNA molecules/µL. A confirmatory *T. cruzi* PCR test was sent to the CDC and was also positive. The patient received benznidazole, and his steroids were discontinued. Repeat *T. cruzi* PCR was negative at follow-up. To date, he has had no long-term sequelae of Chagas disease.

**Figure 4.**
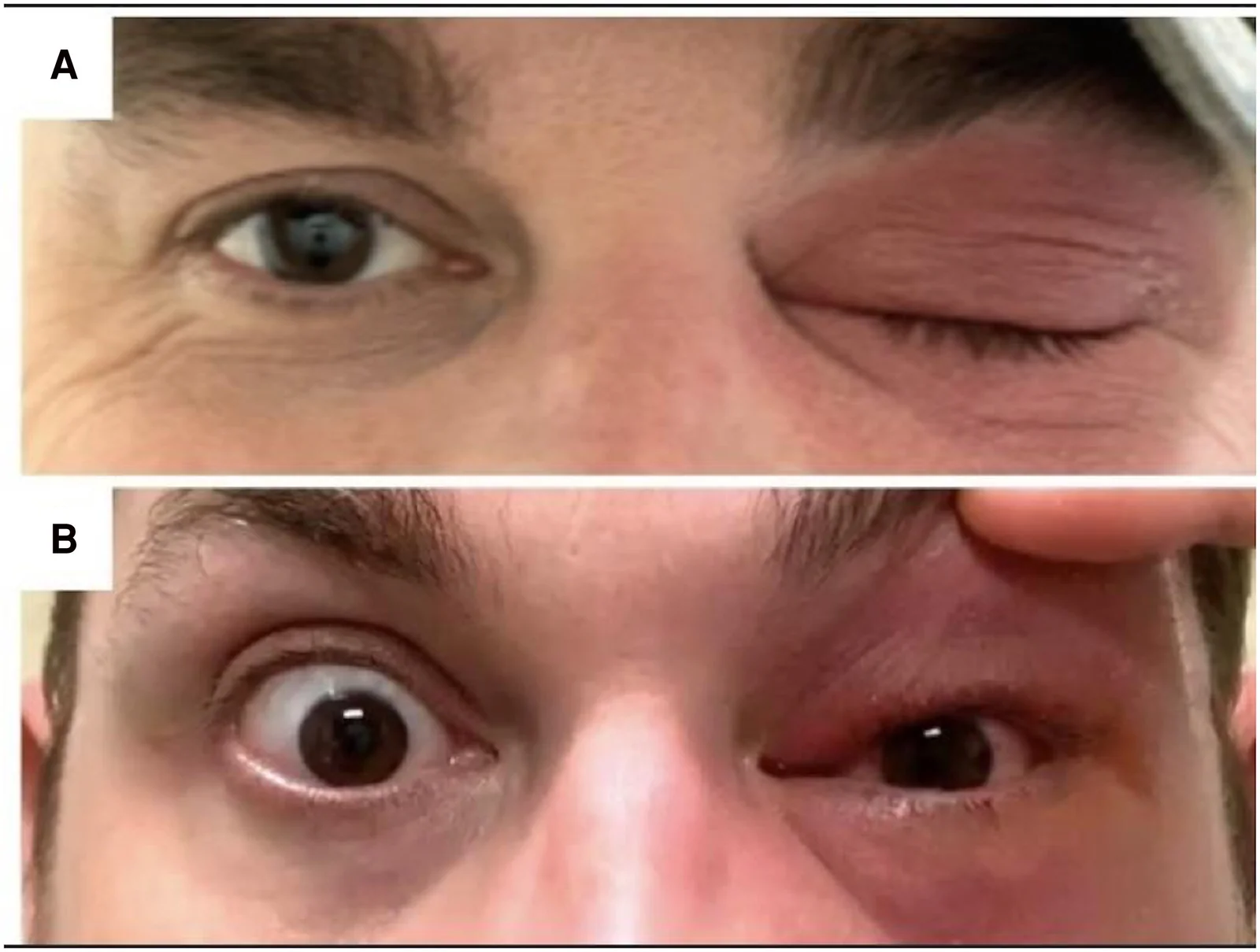
Forty-one-year-old male with Romana’s sign on left eye.

## CASE 7: CARDIOMYOPATHY SECONDARY TO CHAGAS REACTIVATION

A 50-year-old woman with a history of Chagas cardiomyopathy presented with periorbital facial swelling and fevers. She had advanced heart failure and was status post–left ventricular assist device (LVAD) placement as a bridge to heart transplantation. She was highly sensitized against nonself HLA antigens and had received multiple lines of immunosuppressive therapy for desensitization, including bortezomib, obinutuzumab, plasmapheresis, and IVIG. She was receiving daratumumab and concurrent high-dose prednisone at the time of presentation.

She reported waxing and waning left-sided facial and eyelid swelling and erythema for about 5 months prior, which was more persistent over the week before admission. She was febrile on presentation for scheduled daratumumab, with associated fatigue, nausea, and diarrhea in addition to facial swelling. Blood cultures, bacterial and parasitic enteric PCR panel, cytomegalovirus DNA PCR, and COVID-19 testing were sent in addition to 3 sets of parasite smears from PBS. Due to clinical concern for Chagas reactivation, blood was sent for dedicated *T. cruzi* PCR (at the time, the CDC parasitology lab was not accepting specimens; testing was performed through the NY DPH Wadsworth Center laboratory). A Karius test was sent as well due to concerns about potential delays for the dedicated *T. cruzi* PCR test result. The Karius test resulted on day 5 and was positive for *T. cruzi*, while the dedicated PCR resulted on day 10; it was also positive for *T. cruzi*.

She was persistently febrile, and due to delays in acquiring benznidazole, she was started on isavuconazole on day 10, after which she had prompt resolution of fevers and systemic complaints. Benznidazole was acquired and started on day 18 after presentation. Subsequent Karius testing was sent 33 days after presentation, which was negative. She received 29 days of benznidazole; however, treatment was discontinued after she developed oral ulcers and extensive rash involving her palms and soles. Following treatment, she underwent serial monitoring of *T. cruzi* PCR with no subsequent evidence of Chagas reactivation and underwent heart transplantation about 12 months after this episode. She had a complicated post-transplant course characterized by significant bleeding and primary graft dysfunction requiring VA-ECMO support; after recovering from this, she developed a catastrophic central nervous system hemorrhage and died 28 days following transplant.

## DISCUSSION

Our case series highlights the diversity of presentations of acute Chagas disease and how the diagnosis was often not initially considered. The use of plasma mcfDNA testing was a valuable diagnostic tool that provided an accurate and expedited diagnosis of acute Chagas in all patients. Four of the 7 cases involved adult patients who presented with varying symptoms, ranging from right-sided weakness to prolonged respiratory complaints and periorbital erythema with edema. All exhibited fever and clinical signs suggesting an ongoing infectious or inflammatory process. Notably, the patient described as Case 4 had immigrated to the United States from Peru in the 1960s. There was no clinical suspicion of Chagas disease or its reactivation during this time. In addition, the differential diagnosis of encephalitis in adults is often challenging. Often, however, vector-borne diseases are among the main culprits. Reported neurological presentations with Chagas disease include headaches, seizures, and behavioral and muscular disturbances both in the acute and chronic phases [7]. There are classical signs that may raise clinical suspicion for Chagas disease, such as a chagoma, which is a localized inflammatory swelling at the site of parasite entry, and Romaña's sign, which refers to painless periorbital edema resulting from conjunctival inoculation of *Trypanosoma cruzi* [8]. These classical signs are especially seen in Cases 5 and 6, respectively. However, in clinical practice, particularly among those less familiar with the disease, these findings may be overlooked, and the presentation is more commonly mistaken for cellulitis, especially in cases involving periorbital swelling.

Chagas cardiomyopathy has been recognized for many decades as a leading cause of nonischemic cardiomyopathy in Latin America [9]. The United States has witnessed increasing cases due to immigration from different countries in Latin America and Mexico, where Chagas disease is known to be endemic [10]. Recognizing the initial manifestations of Chagas remains a challenge due to limited familiarity with the disease. About 20%–40% of patients who are in the chronic phase are affected by dilated cardiomyopathy [11].

The diagnosis of Chagas disease may not be considered early in infants, as we saw in Case 1, who presented critically ill in heart failure with prolonged fever and findings of HLH. He was treated with empiric antibiotics while undergoing extensive sepsis evaluation in addition to further investigation for the root causes of HLH. Cases 1 and 3 had similar presentations; they both had vague symptoms consisting of febrile episodes that may be seen as upper respiratory infection symptoms. Interestingly, all 3 children were similar in age, ranging from 9 to 12 months old. They shared features that were highly suggestive of infectious myocarditis, and all cases occurred in South Texas. This highlights not only the possibility of congenital transmission but also of local transmission of *T. cruzi* via triatomine insects, which are endemic to the region. It is valuable to screen household members to aid in identifying transmission risks. Because there was no documentation of screening for Chagas within household members for Case 1, a congenital or vector transmission remains in question. There is a possibility of vector transmission that may have occurred when the family resided in Mexico until the child was 3 months of age as it is also known to be an endemic area for Chagas disease. In this case series, testing family members for Chagas was recommended for Cases 2 and 3; they tested negative, which confirms a local transmission rather than a congenital transmission.

Treatment for childbearing women is key to prevent the congenital transmission of Chagas disease. It is critical to prioritize screening of women of childbearing age who present with vague symptoms with known risk factors, such as living in endemic areas. However, patients with Chagas can be asymptomatic, and often it takes about 10–30 years for symptoms to appear. Therefore, treatment is often delayed or not done at all. For the 3 pediatric patients in this case series, there was no suspicion for Chagas disease. A Karius test detected *T. cruzi*. Once the parasite was identified and an antiparasitic drug was initiated, the patient improved significantly.

Similar to the infants, adult patients were started on broad-spectrum antibiotics. Aside from Case 5 and Case 7, Chagas disease was not suspected, and the underlying etiology remained undetermined, prompting a Karius test and a comprehensive diagnostic workup (Table 1). Confirmatory testing revealed positive *T. cruzi* PCR and serologic IgG positivity in 3 of the 4 cases. Noninvasive and invasive testing varied by case, with Case 4 requiring a brain biopsy that demonstrated necrotizing encephalitis with intracellular protozoa. Imaging findings included significant cardiac involvement in Cases 1, 2, and 3 and bilateral cerebral lesions in Case 4. The use of mcfDNA testing in our patients led to an expedited diagnosis of acute Chagas compared with conventional testing, as well as improved antibiotic stewardship and initiation of appropriate targeted therapy. Time to mcfDNA result was reported as ∼2–3 days in Cases 1–5. *T. cruzi* DNA was detected in all patients by Karius testing, with quantifiable levels reported in Cases 2–7 (ranging from 354 to 98 642 DNA molecules/μL). Targeted antitrypanosomal therapy included benznidazole in 6 patients and nifurtimox in 1, administered for ∼60 days when documented.

**Table 1.** Clinical Characteristics, Diagnostic Findings, and Treatment Course of Four Patients Diagnosed With *Trypanosoma cruzi* Infection via Microbial Cell-Free DNA (mcfDNA) Sequencing

	Case 1	Case 2	Case 3	Case 4	Case 5	Case 6	Case 7
Age/gender	12 mo/M	11 mo/M	9 mo/M	88 y/F	71 y/F	41 y/M	50 y/F
Presenting symptoms	Decreased activity, anorexia, cardiomegaly	Cardiac arrest	Fever with concerns for dehydration	Altered mental status, right-sided weakness	Intermittent fever, chills, nonproductive cough, rhinorrhea	Peri-orbital erythema and edema	Peri-orbital swelling, fever
Presence of fever	Yes	No	Yes	Yes	Yes	Yes	Yes
WBC	Elevated	No	Elevated	Elevated	Mildly elevated	Normal	Normal
Blood culture	Negative	Negative	Negative	Negative	Negative	Negative	Negative
Noninvasive diagnostic tests	Prolonged PT/PTT Elevated CRP Significantly elevated ferritin, low fibrinogen, depressed NK cells function Hypoglycemia and severe transaminitis COVID IgM positive EPEC positive in GI PCR	Elevated troponin and BNP levels Elevated CRP and procalcitonin Elevated LDH Peripheral blood smear positive for *Trypanosoma cruzi* Unremarkable infectious labs, such as enterovirus	Leukocyte esterase on UA Elevated procalcitonin and LDH Peripheral blood smear positive for *Trypanosoma cruzi* Elevated PT/INR Transaminitis	Parasitic blood smear negative	Confirmatory *T. cruzi* PCR positive Serologic IgG positive for *T. cruzi* COVID-19 PCR positive Elevated ESR and CRP Peripheral blood smear negative	CMP negative HIV and syphilis negative MRSA negative nasal colonization Negative *Bartonella*, toxoplasmosis, tularemia, and adenovirus	Parasite blood smear negative ×3 COVID-19 PCR negative CMV DNA PCR negative *T. cruzi* DNA PCR from blood positive (d 10 resulted)
Invasive diagnostic tests	CSF grossly unremarkable Bone marrow aspiration positive for organisms consistent with *T. cruzi*	None	None	CSF with pleocytosis and elevated protein CSF PCR negative Brain biopsy demonstrated necrotizing encephalitis and abundant intracellular protozoa	None	None	None
Imaging	Echocardiogram: severe LV dysfunction (ejection fraction of 30%–35%), a thrombus in the left ventricle, and pericardial effusion Head CT: no midline shift or concerns for intraparenchymal hemorrhage	Echocardiogram: worsening pericardial effusion and systolic function with a left ventricular ejection fraction at 45%–49%	Echocardiogram: moderate concentric left ventricle hypertrophy, dyssynchronous ventricular contraction, and small pericardial effusion	Brain MRI: bilateral frontoparietal enhancing white matter changes and surrounding vasogenic edema	Chest X-ray: clear	MRI of the orbit: enhancement of left medial and inferior rectus muscles, compatible with orbital myositis	CT chest, abdomen, pelvis with no acute findings, TTE unchanged from baseline
Empiric antimicrobials	Rocephin Cefepime Doxycycline	None	Ceftriaxone Azithromycin	Initial differentials included TIA vs ADEM, hence empiric antibiotics were not an initial part of treatment	Trimethoprim-sulfamethoxazole	Vancomycin Piperacillin/tazobactam Rifampin Doxycyline	Daptomycin Cefepime
Karius test	*T. cruzi* detected, not quantifiable	95 996 *T. cruzi* DNA molecules/µL 968 DNA molecules/µL Parvovirus B19	98 642 *T. cruzi* DNA/µL	40 000 *T. cruzi* DNA molecules/µL	1379 *T. cruzi* DNA molecules/µL	505 *T. cruzi* DNA molecules/µL	*T. cruzi* detected, 354 DNA molecules/µL
Time to result from mcfDNA collection	2 d	2 d	3 d	3 d	2 d	4 d	5 d
Antibiotics after mcfDNA sequencing	Benznidazole	Benznidazole	Benznidazole	Benznidazole	Nifurtimox (due to unavailability of benznidazole)	Benznidazole	Isavuconazole Benznidazole
Duration of hospitalization	∼35 d	7 d	11 d and was transferred for escalation of care	3 wk	12 d	19 d	20 d
Duration of therapy	60 d	60 d	60 d	60 d	60 d	60 d	Isavuconazole 8 d Benznidazole 29 d

Abbreviations: ADEM, acute demyelinating encephalomyelitis; BNP, brain natriuretic peptide; CMP, complete metabolic panel; CMV, cytomegalovirus; CRP, C-reactive protein; COVID-19, coronavirus disease 2019; CSF, cerebrospinal fluid; CT, computed tomography; EPEC, Enteropathogenic *Escherichia coli*; ESR, erythrocyte sedimentation rate; GI, gastrointestinal; IgM, immunoglobulin M; INR, international normalized ratio; LDH, lactate dehydrogenase; LV, left ventricle; mcfDNA, microbial cell-free DNA; MRI, magnetic resonance imaging; MRSA, methicillin-resistant *Staphylococcus aureus*; NK, natural killer cells; PCR, polymerase chain reaction; PT, prolonged prothrombin time; PTT, partial thromboplastin time; TIA, transient ischemic attack; TTE, transthoracic echocardiogram; UA, urinalysis; WBC, white blood cell count.

At this time, there is no test of cure for Chagas disease. However, most of the patients in this case series were followed after discharge, where *T. cruzi* PCR was collected after treatment completion. Conventional testing is typically performed, as a major limitation of the test remains lack of access in inpatient and outpatient settings due to cost and insurance coverage. Despite increasing endemicity in the United States, there continues to be a knowledge gap about the disease, leading to a delay in recognition. A study has proven that there is a lack of general awareness of Chagas disease within different specialties, with obstetrics and gynecology being of concern based on the possibility of congenital transmission [12]. Therefore, educating health care providers about the disease to establish screening guidelines is critical to overcome this barrier [13]. Given the potential overlap of Chagas disease manifestations with common and complex conditions, the Karius test would alleviate barriers in recognizing the disease and provide a timely and accurate diagnostic advantage, especially in areas with increasing endemicity. In a study that focused on 350 adult patients with sepsis, the Karius test was able to identify a sensitivity of 85% and a specificity of 48% compared with conventional testing, showing its valuable diagnostic advantage in critical settings [14]. Its ability to provide broad pathogen detection with a relatively quick turnaround time also supports earlier diagnosis and targeted therapy. In this case series, the turnaround time for the Karius Spectrum test ranged between 2 and 5 days. This is especially important in complex cases such as those seen in this case series, which included Chagas-induced cardiomyopathy and HLH as well as Chagas reactivation, where traditional diagnostic pathways may delay treatment or lead to misdiagnosis. Prompt use of advanced diagnostics, in conjunction with clinical signs, can facilitate more favorable outcomes and avoid unnecessary empiric treatments. For our patients, the Karius Spectrum provided us with a quick diagnosis when Chagas disease was not initially suspected, allowing us to begin early antiparasitic therapy, discontinue unnecessary antibiotics, and stop the diagnostic workup.
